# Wireless Network Optimization for Federated Learning with Model Compression in Hybrid VLC/RF Systems †

**DOI:** 10.3390/e23111413

**Published:** 2021-10-27

**Authors:** Wuwei Huang, Yang Yang, Mingzhe Chen, Chuanhong Liu, Chunyan Feng, H. Vincent Poor

**Affiliations:** 1 Beijing Key Laboratory of Network System Architecture and Convergence, School of Information and Communication Engineering, Beijing University of Posts and Telecommunications, Beijing 100876, China; wuweihuang@bupt.edu.cn (W.H.); 2016_liuchuanhong@bupt.edu.cn (C.L.); cyfeng@bupt.edu.cn (C.F.); 2 Department of Electrical and Computer Engineering, Princeton University, Princeton, NJ 08544, USA; mingzhec@princeton.edu (M.C.); poor@princeton.edu (H.V.P.)

**Keywords:** federated learning, model compression, visible light communication

## Abstract

In this paper, the optimization of network performance to support the deployment of federated learning (FL) is investigated. In particular, in the considered model, each user owns a machine learning (ML) model by training through its own dataset, and then transmits its ML parameters to a base station (BS) which aggregates the ML parameters to obtain a global ML model and transmits it to each user. Due to limited radio frequency (RF) resources, the number of users that participate in FL is restricted. Meanwhile, each user uploading and downloading the FL parameters may increase communication costs thus reducing the number of participating users. To this end, we propose to introduce visible light communication (VLC) as a supplement to RF and use compression methods to reduce the resources needed to transmit FL parameters over wireless links so as to further improve the communication efficiency and simultaneously optimize wireless network through user selection and resource allocation. This user selection and bandwidth allocation problem is formulated as an optimization problem whose goal is to minimize the training loss of FL. We first use a model compression method to reduce the size of FL model parameters that are transmitted over wireless links. Then, the optimization problem is separated into two subproblems. The first subproblem is a user selection problem with a given bandwidth allocation, which is solved by a traversal algorithm. The second subproblem is a bandwidth allocation problem with a given user selection, which is solved by a numerical method. The ultimate user selection and bandwidth allocation are obtained by iteratively compressing the model and solving these two subproblems. Simulation results show that the proposed FL algorithm can improve the accuracy of object recognition by up to 16.7% and improve the number of selected users by up to 68.7%, compared to a conventional FL algorithm using only RF.

## 1. Introduction

Federated learning (FL), which allows edge devices to cooperatively train a shared machine learning model without transmitting private data, is an emerging distributed machine learning technique [1,2]. The FL training process needs to iteratively transmit machine learning parameters over wireless links. However, due to dynamic wireless channels and imperfect wireless transmission, the performance of FL will be significantly affected by wireless communication. In addition, due to limited communication resources, the number of users that can participate in FL is limited.

### 1.1. Related Work

A number of prior studies in [3,4,5,6,7,8,9,10,11,12,13,14,15,16] have investigated important problems related to wireless network optimization of FL. The works in [3,4,5,6] provided a comprehensive survey of existing studies and summarized open problems in FL. One key challenge is the contradiction between the huge communication costs required by FL parameter transmission and the limited available communication resources [5]. Therefore, on one hand, the existing studies in [7,8,9,10,11,12] proposed to compress the FL model parameters to reduce the communication cost. In particular, the authors of [7] proposed a sparsification and quantization method that compresses the trained FL model. In addition, they also proposed a low rank method and a random mask method, which directly learns a model from a restricted space. In [8], the authors combined quantization and sparsification to implement sketched updates with low sparsity rate. The work in [9] proposed a sparse ternary compression (STC) method that incorporates gradient sparsification, ternary quantization, and lossless encoding, which further improves the compression gains. The authors of [10] proposed to introduce the STC in [9] into structured updates, and thus they focused on compression during the training phase, instead of compressing the trained FL model. Overall, as demonstrated in [7,10], FL model compression can significantly reduce communication costs with minor impact on training accuracy.

On the other hand, the studies in [13,14,15,16] proposed to optimize resource allocation to improve communication efficiency in FL. In [13], the authors studied a joint learning, wireless resource allocation, and user selection optimization problem to improve FL performance. In [14], the trade-off of time and energy consumption, and the trade-off of computational and communication delay have been studied. Meanwhile, the authors of [15] optimized a joint computation and transmission problem, whose goal is to minimize the total energy consumption with communication constraints such as limited computational resources and transmission energy. In addition, the authors of [16] formulated an optimization problem of resource allocation and introduced the use of artificial neural networks (ANNs) to predict the unselected users’ FL model parameters to improve the FL convergence speed and training loss.

Based on the above research, we can observe that the existing studies focus on improving the communication efficiency either by reducing the transmission data amount or optimizing wireless resource allocation to improve the data rate of each user. As a complement of RF, visible light communication (VLC) has advantages including having large, license-free bandwidth, high energy efficiency, and being free of interference to the RF systems. Introducing VLC into FL can significantly supplement the communication resources for FL training. In addition, model compression can be introduced to further reduce the resources needed to transmit FL model parameters and increase the number of users participating in FL training.

### 1.2. Contribution

The main contribution of this paper is a novel hybrid VLC/RF FL algorithm, that jointly optimizes user selection, bandwidth allocation, and model compression (USBA-MC). To the best of our knowledge, this is the first work that introduces the use of VLC techniques for FL performance optimization. The contributions are summarized as follows.

- We propose a USBA-MC algorithm over a hybrid VLC/RF system. In the USBA-MC algorithm, each user obtains a local FL model by training through its own dataset and transmits the model parameters to a base station (BS). The BS aggregates the received local models to generate a global FL model and transmits it back to each user. For the considered FL model, the performance is significantly affected by wireless factors such as available bandwidth and users’ channel state information. This formulates a joint user selection and bandwidth allocation problem, whose goal is to minimize the FL training loss.
- To solve this problem, we first introduce a model compression method to reduce the size of FL model parameters that are transmitted over wireless links. To this end, we first sort the model parameters and design a threshold selection mechanism according to the sparsity rate. Then, we cut off the redundant model parameters based on the threshold and, thus, compress an FL model of each user.
- Following the model compression, we separate the joint user selection and bandwidth allocation problem into two subproblems. The first subproblem is a user selection problem with a given bandwidth allocation, which is solved by a traversal algorithm. The second subproblem is a bandwidth allocation problem with a given user selection, which is solved by a numerical method. The ultimate user selection and bandwidth allocation are obtained by iteratively compressing the model and solving these two subproblems.

Simulation results show that the proposed FL algorithm can improve the accuracy of object recognition by up to 16.7% and improve the number of selected users by up to 68.7%, compared to a conventional FL algorithm using only RF.

The remainder of this paper is organized as follows. In Section 2, we introduce the hybrid VLC/RF system model. Section 3 introduces a model compression method. The joint user selection, bandwidth allocation, and model compression algorithm is described in Section 4. Simulation results are presented and discussed in Section 5. Finally, Section 6 draws some important conclusions.

## 2. System Model and Problem Formulation

In this section, we first introduce a hybrid VLC/RF system for FL. Then, we introduce the computational model and the communication models of RF and VLC systems. Finally, based on the established model, we introduce a user selection and bandwidth allocation problem.

### 2.1. FL Model

In this model, each user *n* stores a local dataset $D_{n}$ with $D_{n}$ being the number of training data samples. Therefore, the total number of training data samples of all users is $D=\sum_{n=1}^{N}D_{n}$. We assume that the training data samples of user *n* can be expressed by $\left\{x_{n},y_{n}\right\}$ with $x_{n}=\left[x_{n1},\ldots ,x_{nD_{n}}\right]$ and $y_{n}=\left[y_{n1},\ldots ,y_{nD_{n}}\right]$, where each $x_{ni}$ is an input vector of the FL algorithm and $y_{ni}$ is the output of $x_{ni}$.

For each user, the FL training purpose is to find the model parameter ω that minimizes the loss function:

(1) $$\begin{array}{c} J_{n}\left(\omega \right):=\frac{1}{D_{n}}\sum_{i\in D_{n}}f_{i}\left(\omega \right), \end{array}$$

where $f_{i}\left(\omega \right)$ is a loss function that captures the performance of the FL algorithm. For example, for a linear regression FL, the loss function is $f_{i}\left(\omega \right)=\frac{1}{2}{\left({x_{i}}^{T}\omega -y_{i}\right)}^{2}$ [14].

All users aim to minimize the following global loss function:

(2) $$\begin{array}{c} \min_{\omega \in R^{d}}J\left(\omega \right):=\sum_{n=1}^{N}\frac{D_{n}}{D}J_{n}\left(\omega \right). \end{array}$$

To solve (2), the BS will first transmit the global FL model parameters to its users, and users will use the received global FL model parameters to train their local FL models. Then, the users will transmit their local FL model parameters to the BS to update the global FL model. For strongly convex objective J(ω), the maximum number of global iterations that an FL algorithm needs to converge is [17]

(3) K(ε,θ)=o(log(1ε))1−θ,

where ε is the accuracy of global model and θ is the accuracy of local model. We consider a fixed global accuracy ε.

### 2.2. FL Based on Hybrid VLC/RF System

**Figure 1:**
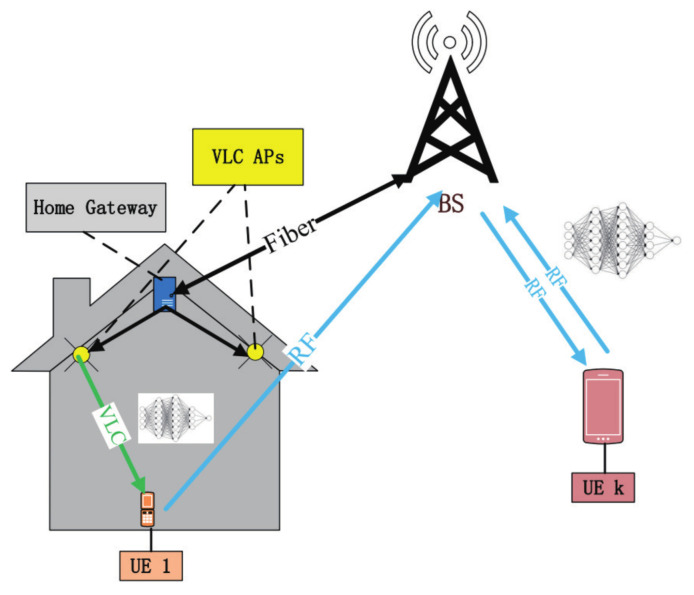
Illustration of FL based on a hybrid VLC/RF system.

Due to the limited wireless bandwidth, only a subset of users can be selected for FL training, which can seriously degrade the training accuracy. To enable more users to join the FL training process, we design a hybrid VLC/RF system. The system structure is shown in Figure 1.

The considered system consists of one BS, home gateways, and users cooperatively performing an FL algorithm for data analysis and inference. Denote the total users by a set N of *N* users. Denote the indoor users by a set $N_{1}$ of $N_{1}$ users and the outdoor users by a set $N_{2}$ of $N_{2}$ users. In this model, the BS will send the global FL model parameters to outdoor users by RF. Meanwhile, the BS transmits the global model parameters to the home gateways which are connected to the indoor VLC access points (APs). Then, the VLC APs transmit the global FL model parameters to indoor users through the visible light signal. Assuming that the BS and home gateways are connected by fiber on which bit errors can be negligible.

In indoor scenarios, each VLC AP consists of an LED lamp. Each user is served by the AP that provides the strongest signal. In addition, we assume that all indoor users can be covered by visible lights. We also assume that there is a central unit (CU) which controls both VLC and RF systems. Note that there is no interference between the RF and VLC systems, which is a key benefit of introducing VLC for the deployment of FL over wireless networks.

### 2.3. Computational Model

Let $c_{n}$ be the number of CPU cycles for user *n* to process one sample of data. As the data size of each training data sample is equal, the number of CPU cycles required for user *n* to execute one local iteration is $c_{n}D_{n}$. Denote the CPU-cycle frequency of user *n* by $f_{n}$. Then, the energy consumption of user *n* updating its local FL model in one global iteration can be expressed as follows:

(4) $$\begin{array}{c} E_{n}^{P}=\frac{\nu \alpha_{n}c_{n}D_{n}}{2}{f_{n}}^{2}\log\left(1/\theta \right), \end{array}$$

where n=1,2,...,N, $\frac{\alpha_{n}}{2}$ is the effective capacitance coefficient of the computing chipset of user *n*, and ν is a positive constant that depends on the data size of training data sample and the number of conditions in the local problem [14].

Furthermore, the computational time per local iteration of user *n* can be denoted as $\frac{c_{n}D_{n}}{f_{n}},n=1,2,...,N$. The computational time, however, depends on the number of local iterations, which is upper bounded by o(log(1/θ)). Therefore, the required computational time of user *n* for data processing is

(5) $$\begin{array}{c} t_{n}^{P}=\frac{\nu c_{n}D_{n}\log\left(1/\theta \right)}{f_{n}}. \end{array}$$

### 2.4. RF Transmission Model

We use the orthogonal frequency division multiple access (OFDMA) technique for both uplink and downlink RF transmissions. The uplink rate of user *n* is given by

(6) $$\begin{array}{c} r_{n}^{U}=\sum_{i=1}^{R^{U}}r_{n,i}^{U}B^{U}\log_{2}\left(1+\frac{P_{n}h_{n}}{\sum_{i^{{}^{\prime }}\in U_{n}^{{}^{\prime }}}P_{i^{{}^{\prime }}}h_{i^{{}^{\prime }}}+B^{U}N_{0}^{R}F}\right), \end{array}$$

where $r_{n}^{U}=\left[r_{n,1}^{U},...,r_{n,R^{U}}^{U}\right]$ is a resource block allocation vector and $R^{U}$ is the total number of RBs that the BS can allocate to the users. $r_{n,i}^{U}\in \left\{0,1\right\}$ and $\sum_{i=1}^{R^{U}}r_{n,i}^{U}=1$; $r_{n,i}^{U}=1$ implies that RB *i* is allocated to user *n*; otherwise, we have $r_{n,i}^{U}=0$; $U_{n}^{{}^{\prime }}$ represents the set of users that are located at the other service areas and transmit data over RB *i*; $B^{U}$ is the bandwidth of each RB and $P_{n}$ is the transmit power of user *n*; $h_{n}$ is the channel gain between user *n* and the BS; $N_{0}^{R}F$ is the noise power spectral density; $\sum_{i^{{}^{\prime }}\in U_{n}^{{}^{\prime }}}P_{i^{{}^{\prime }}}h_{i^{{}^{\prime }}}$ is the interference caused by the users that are located in other service areas and use the same RB.

On the other hand, the downlink data rate of the BS transmitting global FL model parameters to each user *n* is given by

(7) $$\begin{array}{c} r_{n}^{D}=\sum_{i=1}^{R^{D}}r_{n,i}^{D}B^{D}\log_{2}\left(1+\frac{P_{B}h_{n}}{\sum_{j\in B^{{}^{\prime }}}P_{B}h_{nj}+B^{D}N_{0}^{R}F}\right), \end{array}$$

where $B^{D}$ is the bandwidth of each RB that the BS used to transmit the global FL model to each user *n*; $r_{n}^{D}=\left[r_{n,1}^{D},...,r_{n,R^{D}}^{D}\right]$ is a RB allocation vector with $R^{D}$ being the total number of RBs that the BS can be used for FL parameter transmission. $r_{n,i}^{D}\in \left\{0,1\right\}$ and $\sum_{i=1}^{R^{D}}r_{n,i}^{D}=1$; $r_{n,i}^{D}=1$ indicates that RB *i* is allocated to user *n*; otherwise, we have $r_{n,i}^{D}=0$; $P_{B}$ is the transmit power of the BS; $B^{{}^{\prime }}$ is the set of other BSs that cause interference to the BS that performs the FL algorithm; $h_{nj}$ is the channel gain between user *n* and BS *j*. Let $B_{R}$ be the total RF bandwidth, and we have $R^{U}\times B^{U}+R^{D}\times B^{D}\leq B_{R}$. For simplicity, we assume $B^{U}=B^{D}$ which means the bandwidth of an uplink resource block is equal to that of a downlink RB.

Denote the data size in bit of an FL model that each user needs to upload by $s^{L}$. To upload the local FL model within transmission delay requirement $t_{n}^{U}$, we have $t_{n}^{U}r_{n}^{U}\geq s^{L}$. Meanwhile, the required energy of user *n* transmitting FL parameters is $E_{n}^{M}=t_{n}^{U}P_{n}$. Similarly, we assume that the data size in bit of the global parameters which are transmitted to users is $s^{G}$. To download the global FL model within transmission delay $t_{n}^{D}$, we have $t_{n}^{D}r_{n}^{D}\geq s^{G}$.

### 2.5. VLC Transmission Model

The optical channel gain of a line-of-sight (LoS) channel can be expressed as [18]

(8) $$\begin{array}{c} u=\left\{\begin{array}{c} \frac{\left(m+1\right)A_{p}}{2\pi d^{2}}T_{s}\left(\theta \right)g\left(\theta \right)\cos^{m}\left(\varphi \right)\cos\left(\theta \right),0<\theta \leq \Theta_{F},\\ 0,\theta >\Theta_{F}, \end{array}\right. \end{array}$$

where $m=-\frac{1}{\log_{2}\left(\cos\left(\theta_{1/2}\right)\right)}$ is the Lambertian index which is a function of the half-intensity radiation angle $\theta_{1/2}$, $A_{p}$ is the receiver’s physical area of the photo-diode, *d* is the distance from the VLC AP to the optical receiver, φ is the angle of irradiation and θ is the angle of incidence, $\Theta_{F}$ is the half angle of the receiver’s file of view (FoV), $T_{s}\left(\theta \right)$ is the gain of the optical filter, and the concentrator gain g(θ) can be written as

(9) $$\begin{array}{c} g\left(\theta \right)=\left\{\begin{array}{c} \frac{n_{0}^{2}}{\sin^{2}\Theta_{F}},0<\theta \leq \Theta_{F},\\ 0,\theta >\Theta_{F}, \end{array}\right. \end{array}$$

where $n_{0}$ is the refractive index. For a given user *n* connected to a VLC AP *k*, the signal-to-interference-plus-noise ratio (SINR) can be written as

(10) $$\begin{array}{c} s_{nk}=\frac{{\left(\gamma u_{nk}P_{v}\right)}^{2}}{N_{0}^{V}LCB+\sum_{l\neq k}{\left(\gamma u_{nl}P_{v}\right)}^{2}}, \end{array}$$

where γ is the optical to electric conversion efficiency, $P_{v}$ is the transmitted optical power of a VLC AP, $N_{0}^{VLC}$ is the noise power spectral density, $u_{nk}$ is the channel gain between user *n* and the VLC AP *k*, $u_{nl}$ is the channel gain between user *n* and the interfering VLC AP *l*, and *B* is the bandwidth of each VLC RB. Each user is served by a single VLC AP which has the largest SINR for the user. In the VLC systems, optical OFDMA is employed. It is known that the input signal of the LEDs is amplitude constrained. Therefore, the classical Shannon capacity formula for complex and average power constrained signal is not applicable in VLC. Therefore, the lower bound of achievable data rate is used, which can be expressed as [19]

(11) $$\begin{array}{c} r_{n}=\sum_{i=1}^{R^{V}}r_{n,i}^{V}\frac{B}{2}\log_{2}\left(1+\frac{2}{\pi e}s_{n}\right), \end{array}$$

where $s_{n}$ is the largest SINR which is evaluated as $s_{n}=\max\left\{s_{n1},...,s_{nK}\right\}$, where *K* is the total number of VLC APs; $r_{n}^{V}=\left[r_{n,1}^{V},...,r_{n,R^{V}}^{V}\right]$ is a RB allocation vector with $R^{V}$ being the total number of VLC RBs, $r_{n,i}^{V}\in \left\{0,1\right\}$ and $\sum_{i=1}^{R^{V}}r_{n,i}^{V}=1$; $r_{n,i}^{V}=1$ indicates that RB *i* is allocated to user *n*; otherwise, we have $r_{n,i}^{V}=0$. Similarly, we have: $R^{V}\times B\leq B_{V}$, where $B_{V}$ is the total bandwidth of VLC.

As the data size of global parameters is $s^{G}$, the downlink transmission delay of indoor user *n* in each global iteration will be $t_{dn}=\frac{s^{G}}{r_{n}}$.

### 2.6. Problem Formulation

Next, we introduce the optimization problem. Our goal is to minimize the global loss function under time, energy, and bandwidth allocation constraints. The minimization problem is given by

(12) $$\begin{array}{cc} \; & \min_{B,B^{D},B^{U},S}J\left(\omega \right) \end{array}$$

(12a) $$\begin{array}{cc} s.\;t.\;\;\; & R^{U}\times B^{U}+R^{D}\times B^{D}\leq B_{R}, \end{array}$$

(12b) $$\begin{array}{cc} \; & R^{V}\times B\leq B_{V}, \end{array}$$

(12c) $$\begin{array}{cc} \; & t_{dn}+t_{n}^{U}+t_{n}^{P}+t_{d}\leq T_{round,}\forall n\in S_{1}, \end{array}$$

(12d) $$\begin{array}{cc} \; & t_{n}^{D}+t_{n}^{U}+t_{n}^{P}\leq T_{round,}\forall n\in S_{2}, \end{array}$$

(12e) $$\begin{array}{cc} \; & S_{1}\cup S_{2}=S, \end{array}$$

(12f) $$\begin{array}{cc} \; & E_{n}^{M}+E_{n}^{P}\leq \gamma_{nE},\forall n\in N, \end{array}$$

(12g) $$\begin{array}{cc} \; & R^{U}=\left|S\right|,R^{D}=\left|S_{2}\right|,R^{V}=\left|S_{1}\right|, \end{array}$$

where S denotes the set of selected users participating in FL, $S_{1}$ denotes the set of selected indoor users, $S_{2}$ denotes the set of selected outdoor users, and $\left|.\right|$ denotes the cardinality of a set. In addition, $T_{round}$ is the time threshold for each round and $t_{d}$ denotes the delay between BS and the home gateway. In addition, $\gamma_{nE}$ is the energy constraint of user *n*. (12a) and (12b) are the bandwidth constraints of RF link and VLC link, respectively. Constraint (12c) is the delay constraint of each round for all selected indoor users while (12d) is the delay constraint of each round for all selected outdoor users. (12e) denotes the set of selected users. In addition, (12f) is the energy consumption requirement of performing an FL algorithm.

## 3. Model Compression

In this section, we first analyze the optimization problem (12) so as to figure out how the communication factors affect the FL performance. Then, we introduce a model compression method to reduce the size of FL model parameters that are transmitted over wireless links so as to increase the number of users that participate in FL.

### 3.1. Problem Analysis

To simplify problem (12), we first provide the following lemma:

**Lemma** **1.**  *Given the transmit power of each user, the optimization problem (12) can be transformed into an optimization problem aiming to maximize the total size of data samples of the selected users, which can be denoted as*

(13) $$\begin{array}{cc} \; & \max_{B,B^{D},B^{U},S}\sum_{n=1}^{N_{1}}\sum_{i=1}^{R^{V}}D_{n}r_{n,i}^{V}+\sum_{n=N_{1}+1}^{N}\sum_{i=1}^{R^{D}}D_{n}r_{n,i}^{D} \end{array}$$

(13a) $$\begin{array}{cc} s.\;t.\;\;\; & R^{U}\times B^{U}+R^{D}\times B^{D}\leq B_{R}, \end{array}$$

(13b) $$\begin{array}{cc} \; & R^{V}\times B\leq B_{V}, \end{array}$$

(13c) $$\begin{array}{cc} \; & t_{dn}+t_{n}^{U}+t_{n}^{P}+t_{d}\leq T_{round,}\forall n\in S_{1}, \end{array}$$

(13d) $$\begin{array}{cc} \; & t_{n}^{D}+t_{n}^{U}+t_{n}^{P}\leq T_{round,}\forall n\in S_{2}, \end{array}$$

(13e) $$\begin{array}{cc} \; & S_{1}\cup S_{2}=S, \end{array}$$

(13f) $$\begin{array}{cc} \; & E_{n}^{M}+E_{n}^{P}\leq \gamma_{nE},\forall n\in N, \end{array}$$

(13g) $$\begin{array}{cc} \; & R^{U}=\left|S\right|,R^{D}=\left|S_{2}\right|,R^{V}=\left|S_{1}\right|, \end{array}$$

**Proof.**  Minimizing the global loss function is equivalent to minimizing the gap between the global loss function $J(\omega_{t})$ at time *t* and the optimal global loss function $J(\omega^{*})$. According to the Theorem 1 in [13], the gap is caused by the packet error rate (PER) and the number of selected users. Here, we do not consider the packet errors and hence, we have $q_{i}=0$. Using the same simplification method in [13], the optimization problem can be transformed to problem (13). This ends the proof.    □

### 3.2. Model Weights Compression

From problem (13), we observe that as the number of users that implement FL increases, the gap decreases, and the performance is improved. This is coincide with the experimental conclusions in [20]. To maximize the number of users in FL, we introduce a model compression method to reduce the transmission delay, energy, and bandwidth, so as to increase the number of users that participate in FL. In particular, the FL model has data redundancy during training and, thus, we prune the connections with small weight updates to reduce the size of transmission model parameters. Meanwhile, although the model compression will lose a part of model information, the experiments in [9,10,21] have proved that appropriate compression methods do not significantly affect the convergence speed and accuracy under proper sparsity rates. In this section, we first introduce a compression method with non-fixed thresholds. Then, we analyze the impact of the model compression on the optimization problem (13).

An FL model needs to be carefully compressed without affecting the global model training. The change of weights in a model can be used to evaluate their importance [22]. Therefore, an appropriate pruning threshold is the key for FL model compression. To ensure that the gradients of an FL model are in the same order, we first normalize the gradients in each layer [23]. In particular, the gradients of an FL model can be given by

(14) $$G_{n}^{\tau }=Train\left(W_{n}^{\tau },D_{n}\right)-W_{n}^{\tau },$$

where $G_{n}^{\tau }\in R^{d_{1}\times d_{2}}$ is the gradients of user *n* at iteration τ, $W_{n}^{\tau }\in R^{d_{1}\times d_{2}}$ is the trained local model weights, and τ∈{1,…,T} is a global iteration; $d_{1}$ and $d_{2}$ represent the output and input dimensions, respectively; and $Train(W_{n}^{\tau },D_{n})$ refers to the trained model weights of user *n*. For a given sparsity rate, we obtain a threshold according to the sorted gradients. In particular, the weights less than the threshold are set to 0, while those larger than the threshold are set to 1. This process can be expressed by a sparsifying filter mask $M_{n}\in R^{d_{1}\times d_{2}}$ for user *n*. Therefore, the compressed local model weights can be written as

(15) $$W_{n,C}=W_{n}⊗M_{n},$$

where $W_{n}\in R^{d_{1}\times d_{2}}$ is the local model weights of user *n* and ⊗ is the Hadamard product. Similarly, the compressed global model weights can be expressed as

(16) $$W_{C}=W⊗M,$$

where $W\in R^{d_{1}\times d_{2}}$ is the global model weights and $M\in R^{d_{1}\times d_{2}}$ is the sparsifying filter mask for the BS. From (16), we observe that each user receives the same sparse global model.

The gradients that are not transmitted to the BS or the users are called residuals [24], which will be used for the local model training and the global FL model generation. Therefore, residuals can be used to mitigate the errors caused by the sparsification and accelerate the FL convergence speed [21]. In particular, the residuals of user *n* can be defined by

(17) $$R_{n}^{T}=\sum_{\tau =1}^{T}\left(G_{n}^{\tau }-G_{n,C}^{\tau }\right)=R_{n}^{T-1}+G_{n}^{T}-G_{n,C}^{T},$$

where $R_{n}^{T}\in R^{d_{1}\times d_{2}}$ is the accumulation of the residuals at iteration *T*, and $G_{n,C}^{\tau }\in R^{d_{1}\times d_{2}}$ denotes compressed $G_{n}^{\tau }$. Similarly, residuals of the BS can be defined by

(18) $$R^{T}=\sum_{\tau =1}^{T}\left(G^{\tau }-G_{C}^{\tau }\right)=R^{T-1}+G^{T}-G_{C}^{T},$$

where $G^{\tau }\in R^{d_{1}\times d_{2}}$ is the model gradients at the BS, and $G_{C}^{\tau }\in R^{d_{1}\times d_{2}}$ is the compressed $G^{\tau }$.

During transmissions, users only need to transmit the positions of non-zero parameters and their values. Through receiving these information, the BS can recover the model, and get the sparsifying filter masks. We assume the initial model weights is $W^{I}\in R^{d_{1}\times d_{2}}$, the final output is $W^{F}\in R^{d_{1}\times d_{2}}$ and the matrix with all elements of 1 is 1. The overall process of model compression is shown in Algorithm 1.

Let $p_{n}$ and *p* be the sparsity rate corresponding to $M_{n}$ and M. Then, the size of the compressed local FL model and the compressed global FL model can be expressed as $s_{C}^{L}=s^{L}\cdot p_{n}$ and $s_{C}^{G}=s^{G}\cdot p$, respectively. Using the proposed compression scheme, the optimization problem (13) can be rewritten by

(19) $$\begin{array}{cc} \; & \max_{B,B^{D},B^{U},S}\sum_{n=1}^{N_{1}}\sum_{i=1}^{R^{V}}D_{n}r_{n,i}^{V}+\sum_{n=N_{1}+1}^{N}\sum_{i=1}^{R^{D}}D_{n}r_{n,i}^{D} \end{array}$$

(19a) $$\begin{array}{cc} s.\;t.\;\;\; & R^{U}\times B^{U}+R^{D}\times B^{D}\leq B_{R}, \end{array}$$

(19b) $$\begin{array}{cc} \; & R^{V}\times B\leq B_{V}, \end{array}$$

(19c) $$\begin{array}{cc} \; & t_{dn,C}+t_{n,C}^{U}+t_{n}^{P}+t_{d,C}\leq T_{round,}\forall n\in S_{1}, \end{array}$$

(19d) $$\begin{array}{cc} \; & t_{n,C}^{D}+t_{n,C}^{U}+t_{n}^{P}\leq T_{round,}\forall n\in S_{2}, \end{array}$$

(19e) $$\begin{array}{cc} \; & S_{1}\cup S_{2}=S, \end{array}$$

(19f) $$\begin{array}{cc} \; & E_{n,C}^{M}+E_{n}^{P}\leq \gamma_{nE},\forall n\in N, \end{array}$$

(19g) $$\begin{array}{cc} \; & R^{U}=\left|S\right|,R^{D}=\left|S_{2}\right|,R^{V}=\left|S_{1}\right|, \end{array}$$

where $t_{dn,C}=\frac{s_{C}^{G}}{r_{n}}$, $t_{n,C}^{U}=\frac{s_{C}^{L}}{r_{n}^{U}}$, $t_{n,C}^{D}=\frac{s_{C}^{G}}{r_{n}^{D}}$ and $E_{n,C}^{M}=t_{n,C}^{U}\cdot P_{n}$. Accordingly, we denote the compression algorithm that reduces the communication costs in each iteration by **MC** ($s^{L},s^{G}$).
**Algorithm 1**: FL Model Compression1:**Input:**$W^{I}$2:**for**τ∈{1,…,T}**do**3:  **for** n∈{1,…,N} **do**4:    **Client n does:**5:    **$\left(W_{C}^{t-1},M\right)\leftarrow Download_{BS\to n}\left(W_{C}^{t-1}\right)$**
6:    **$W_{n}^{t}\leftarrow W_{C}^{t-1}+\left(1-M\right)⊗W_{n}^{t-1}$**
7:    **$G_{n}^{t}\leftarrow Train\left(W_{n}^{t},D_{n}\right)+R_{n}^{t-1}-W_{n}^{t}$**
8:    **$M_{n}\leftarrow Compress\left(G_{n}^{t}\right)$**
9:    **$G_{n,C}^{t}\leftarrow G_{n}^{t}⊗M_{n}$**
10:    **$W_{n,C}^{t}\leftarrow W_{n}^{t}⊗M_{n}$**
11:    **$R_{n}^{t}\leftarrow G_{n}^{t}-G_{n,C}^{t}$**
12:    **$Save(R_{n}^{t},W_{n}^{t})$**
13:    **$Upload_{n\to BS}\left(W_{n,C}^{t}\right)$**
14:  **end for**15:  **BS does:**16:  **for** $n\in S=S_{1}\cup S_{2}$ **do**17:    **$\left(W_{n,C}^{t},M_{n}\right)\leftarrow W_{n,C}^{t}$**
18:  **end for**19:  **$W_{C}^{t}\leftarrow Aggregate\left(\frac{1}{\left|S\right|}\sum_{n\in S}W_{n,C}^{t}\right)+R^{t-1}$**
20:  **$W^{t}\leftarrow W_{C}^{t}+\left(1-M_{1}\right)⊗\left(1-M_{2}\right)\cdots ⊗\left(1-M_{n}\right)⊗W^{t-1}$**
21:  **$G^{t}\leftarrow W^{t}-W^{t-1}$**
22:  **$M\leftarrow Compress(G^{t})$**
23:  **$G_{C}^{t}\leftarrow G^{t}⊗M$**
24:  **$W_{C}^{t}\leftarrow W^{t}⊗M$**
25:  **$R^{t}\leftarrow G^{t}-G_{C}^{t}$**
26:  **$Save(R^{t},W^{t})$**
27:  **$Transmits_{BS\to n}\left(W_{C}^{t}\right)$**
28:**end for**29:**Return**$W^{F}$

## 4. The Proposed Algorithm

To solve (19), in this section, we propose a joint user selection, bandwidth allocation, and model compression algorithm, USBA-MC, which divides problem (19) into two subproblems and solve them iteratively. In particular, we first fix the bandwidth allocation and optimize user selection. Then, the problem of bandwidth allocation is formulated and solved with the obtained subset of the selected users. The model compression and these subproblems are iteratively solved until a convergent solution of (19) is obtained.

### 4.1. Optimal User Selection

Given the bandwidth of each RB, (19) can be further simplified as

(20) $$\begin{array}{cc} \; & \max_{S}\sum_{n=1}^{N_{1}}\sum_{i=1}^{R^{V}}D_{n}r_{n,i}^{V}+\sum_{n=N_{1}+1}^{N}\sum_{i=1}^{R^{D}}D_{n}r_{n,i}^{D} \end{array}$$

(20a) $$\begin{array}{cc} s.\;t.\;\; & t_{dn,C}+t_{n,C}^{U}+t_{n}^{P}+t_{d,C}\leq T_{round,}\forall n\in S_{1}, \end{array}$$

(20b) $$\begin{array}{cc} \; & t_{n,C}^{D}+t_{n,C}^{U}+t_{n}^{P}\leq T_{round,}\forall n\in S_{2}, \end{array}$$

(20c) $$\begin{array}{cc} \; & S_{1}\cup S_{2}=S, \end{array}$$

(20d) $$\begin{array}{cc} \; & E_{n,C}^{M}+E_{n}^{P}\leq \gamma_{nE},\forall n\in N, \end{array}$$

(20e) $$\begin{array}{cc} \; & R^{U}=\left|S\right|,R^{D}=\left|S_{2}\right|,R^{V}=\left|S_{1}\right|. \end{array}$$

We can observe from (20) that if the bandwidth of each RB is fixed, the subset of selected users is determined by the users’ computing power and channel condition. We denote the algorithm that optimizes user selection under fixed bandwidth allocation by Algorithm 2.
**Algorithm 2**: User Selection Algorithm **GetS**($B^{U}$,$B^{D}$,*B*)1:**Input:**$N_{1},N_{2}$2:**for**$n\in N_{1}$**do**3:  **if** $t_{dn,C}+t_{n,C}^{U}+t_{n}^{P}+t_{d,C}\leq T_{round,}$ **and** $E_{n,C}^{M}+E_{n}^{P}\leq \gamma_{nE}$ **then**4:    $S_{1}\leftarrow n$5:  **end if**6:**end for**7:**for**$n\in N_{2}$**do**8:  **if** $t_{n,C}^{D}+t_{n,C}^{U}+t_{n}^{P}\leq T_{round,}$ **and** $E_{n,C}^{M}+E_{n}^{P}\leq \gamma_{nE}$ **then**9:   $S_{2}\leftarrow n$10:  **end if**11:**end for**

### 4.2. Optimal RB Bandwidth

With an obtained subset of users, we need to find the optimal *B*, $B^{U}$, and $B^{D}$ that can further optimize the capability of the hybrid VLC/RF systems. Note that the larger the bandwidth of a RB is, the smaller the delay can be, implying more users can be potentially selected. Based on this observation, the optimal RB bandwidth allocation problems are

(21) $$\begin{array}{cc} \; & \max B^{U} \end{array}$$

(21a) $$\begin{array}{cc} s.\;t.\;\; & R^{U}\times B^{U}+R^{D}\times B^{D}\leq B_{R}, \end{array}$$

(21b) $$\begin{array}{cc} \; & B^{U}=B^{D}, \end{array}$$

(21c) $$\begin{array}{cc} \; & R^{U}=\left|S\right|,R^{D}=\left|S_{2}\right|, \end{array}$$

and

(22) $$\begin{array}{cc} \; & \max B \end{array}$$

(22a) $$\begin{array}{cc} \mathrm{s. t.}\;\; & R^{V}\times B\leq B_{V}, \end{array}$$

(22b) $$\begin{array}{cc} \; & R^{V}=\left|S_{1}\right|. \end{array}$$

**Lemma** **2.**  *The maximum bandwidth of a RB can be obtained when $R^{U}\times B^{U}+R^{D}\times B^{D}=B_{R}$ and $R^{V}\times B=B_{V}$.*

**Proof.**  We use the contradiction method to prove *Lemma* 2. First, we assume that maximum $B_{0}^{U}$, $B_{0}^{D}$, and $B_{0}$ exist when (21a) and (22a) are not equal. Therefore, we have

(23) $$\begin{array}{c} B_{0}^{U}=B_{0}^{D}<\frac{B_{R}}{\left|S\right|+\left|S_{2}\right|}, \end{array}$$

and

(24) $$\begin{array}{c} B_{0}<\frac{B_{V}}{\left|S_{1}\right|}. \end{array}$$

However, when (21a) and (22a) are equal, $B_{1}^{U}$, $B_{1}^{D}$, and $B_{1}$ satisfy the following equations:

(25) $$\begin{array}{c} B_{1}^{U}=B_{1}^{D}=\frac{B_{R}}{\left|S\right|+\left|S_{2}\right|}, \end{array}$$

and

(26) $$\begin{array}{c} B_{1}=\frac{B_{V}}{\left|S_{1}\right|}. \end{array}$$

Obviously, $B_{1}^{U}=B_{1}^{D}>B_{0}^{U}=B_{0}^{D}$ and $B_{1}>B_{0}$, which contradicts the assumption. This ends the proof.    □

Therefore, we have

(27) $$\begin{array}{c} B^{U}=B^{D}=\frac{B_{R}}{R^{U}+R^{D}}=\frac{B_{R}}{\left|S\right|+\left|S_{2}\right|}, \end{array}$$

and

(28) $$\begin{array}{c} B=\frac{B_{V}}{R^{V}}=\frac{B_{V}}{\left|S_{1}\right|}. \end{array}$$

### 4.3. Iterative Solution

In each iteration, we first use the proposed model compression method to reduce the transmission delay and energy. Then, we update the selected users based on the constraints, using **GetS**($B^{U}$,$B^{D}$,*B*). Finally, the bandwidth allocation is obtained by the given selected users, which is denoted by **GetB**(S). The iteration ends when both the user selection and bandwidth allocation remain fixed. Obviously, the algorithm can always reach convergence after a certain number of iterations. We summarize the proposed USBA-MC algorithm in Algorithm 3.
**Algorithm 3**: USBA-MC Algorithm1:**Input:**$B_{0},B_{0}^{D},B_{0}^{U}$2:**$\left(t_{n,C}^{U},t_{n,C}^{D},t_{dn,C},E_{n,C}^{M}\right)\leftarrow \mathrm{MC}\left(s^{L},s^{G}\right)$**3:$S^{0}\leftarrow$**GetS**($B_{0}^{U},B_{0}^{D},B_{0}$)4:**for**τ∈{1,…,T}**do**5:   $(B_{\tau }^{U},B_{\tau }^{D},B_{\tau })\leftarrow$**GetB**($S^{\tau -1}$)6:   **$\left(t_{n,C}^{U},t_{n,C}^{D},t_{dn,C},E_{n,C}^{com}\right)\leftarrow \mathrm{MC}\left(s^{L},s^{G}\right)$**
7:   $S^{\tau }\leftarrow$**GetS**($B_{\tau }^{U},B_{\tau }^{D},B_{\tau }$)8:   **if** $S^{\tau }==S^{\tau -1}$ and $\left(B_{\tau }^{U},B_{\tau }^{D},B_{\tau }\right)==\left(B_{\tau -1}^{U},B_{\tau -1}^{D},B_{\tau -1}\right)$ **then**9:     **break**10:   **end if**11:**end for**

### 4.4. Convergence, Implementation, and Complexity Analysis

(1) *Convergence Analysis*: We first analyze the convergence of the proposed algorithm. Let the indoor user selection vector be $s_{1}=\left[s_{1}^{1},\ldots ,s_{1}^{N}\right]$ and outdoor user selection vector be $s_{2}=\left[s_{2}^{1},\ldots ,s_{2}^{N}\right]$, where $s_{1}^{n}=1/s_{2}^{n}=1$ indicates user *n* performs the FL algorithm; otherwise, we have $s_{1}^{n}=0/s_{2}^{n}=0$. Assume that the gradient $\nabla J(\omega (s_{1},s_{2}))$ of $J(\omega (s_{1},s_{2}))$ is uniformly Lipschitz continuous with respect to $\omega (s_{1},s_{2})$ [25]. Therefore, we have

(29) $$||\nabla J\left(\omega_{t+1}\left(s_{1},s_{2}\right)\right)-\nabla J\left(\omega_{t}\left(s_{1},s_{2}\right)\right)\left|\right|\leq L||\omega_{t+1}\left(s_{1},s_{2}\right)-\omega_{t}\left(s_{1},s_{2}\right)\left|\right|,$$

where $\omega_{t}\left(s_{1},s_{2}\right)$ is the global model at step *t*, *L* is a positive constant, and ||·|| denotes the norm. Assume that $J(\omega (s_{1},s_{2}))$ is strongly convex with positive parameter μ. Therefore, we have

(30) $$\begin{array}{c} J\left(\omega_{t+1}\left(s_{1},s_{2}\right)\right)\geq J\left(\omega_{t}\left(s_{1},s_{2}\right)\right)+{\left(\omega_{t+1}\left(s_{1},s_{2}\right)-\omega_{t}\left(s_{1},s_{2}\right)\right)}^{T}\nabla J\left(\omega_{t}\left(s_{1},s_{2}\right)\right)\\ +\frac{\mu }{2}\left|\right|\omega_{t+1}\left(s_{1},s_{2}\right)-\omega_{t}\left(s_{1},s_{2}\right){\left|\right|}^{2}. \end{array}$$

We also assume that $J(\omega (s_{1},s_{2}))$ is twice-continuously differentiable. Moreover, we assume $||\nabla f_{i}\left(\omega_{t}\left(s_{1},s_{2}\right)\right)\left|\right|\leq \vartheta_{1}+\vartheta_{2}\nabla ||J\left(\omega_{t}\left(s_{1},s_{2}\right)\right){\left|\right|}^{2}$ with $\vartheta_{1}\geq 0$ and $\vartheta_{2}\geq 0$. The above assumptions are easy to satisfy, such as the loss function that is linear or logistic regression [25]. The expected convergence rate of the proposed algorithm can be obtained by the following lemma:

**Lemma** **3.**  *Given the optimal global FL model $\omega^{*}$. The convergent upper bound of $E[J\left(\omega_{t+1}\left(s_{1},s_{2}\right)\right)-J\left(\omega^{*}\right)]$ applicable to the considered hybrid VLC/RF system satisfies*

(31) $$\begin{array}{c} E\left[J\left(\omega_{t+1}\left(s_{1},s_{2}\right)\right)-J\left(\omega^{*}\right)\right]\leq \frac{2\vartheta_{1}}{LD}(\sum_{n=1}^{N_{1}}\sum_{i=1}^{R^{V}}D_{n}\left(1-r_{n,i}^{V}\right)\\ +\sum_{n=N_{1}+1}^{N}\sum_{i=1}^{R^{D}}D_{n}\left(1-r_{n,i}^{D}\right))\frac{1}{1-F}, \end{array}$$

*where $F=1-\frac{\mu }{L}+\frac{4\mu \vartheta_{2}}{LD}\left(\sum_{n=1}^{N_{1}}\sum_{i=1}^{R^{V}}D_{n}\left(1-r_{n,i}^{V}\right)+\sum_{n=N_{1}+1}^{N}\sum_{i=1}^{R^{D}}D_{n}\left(1-r_{n,i}^{D}\right)\right)$.*

**Proof.**  As $s_{1}^{n}+s_{2}^{n}=\left\{\begin{array}{cc} 1, & \begin{array}{ccc} s_{1}^{n}=1,s_{2}^{n}=0 & \mathrm{or} & s_{1}^{n}=0,s_{2}^{n}=1 \end{array}\\ 0, & \begin{array}{c} s_{1}^{n}=0,s_{2}^{n}=0 \end{array} \end{array}\right.$, we have $1-\left(s_{1}^{n}+s_{2}^{n}\right)=\left\{\begin{array}{cc} 0, & \begin{array}{ccc} s_{1}^{n}=1,s_{2}^{n}=0 & \mathrm{or} & s_{1}^{n}=0,s_{2}^{n}=1 \end{array}\\ 1, & \begin{array}{c} s_{1}^{n}=0,s_{2}^{n}=0 \end{array} \end{array}\right.$. Therefore, we have $1-(s_{1}^{n}+s_{2}^{n})\geq 0$ with n=[1,...,N]. Then, the upper bound of $E[J\left(\omega_{t+1}\left(s_{1},s_{2}\right)\right)-J\left(\omega^{*}\right)]$ can be obtained according to the Theorem 1 in [13] as

(32) $$\begin{array}{c} E\left[J\left(\omega_{t+1}\left(s_{1},s_{2}\right)\right)-J\left(\omega^{*}\right)\right]\leq F^{t}E\left[J\left(\omega_{0}\right)-J\left(\omega^{*}\right)\right]\\ +\frac{2\vartheta_{1}}{LD}\sum_{n=1}^{N}D_{n}\left(1-\left(s_{1}^{n}+s_{2}^{n}\right)\right)\frac{1-F^{t}}{1-F}, \end{array}$$

where $F=1-\frac{\mu }{L}+\frac{4\mu \vartheta_{2}}{LD}\sum_{n=1}^{N}D_{n}\left(1-\left(s_{1}^{n}+s_{2}^{n}\right)\right)$. As each resource block will be assigned to a participated user, the upper bound can be further converted into

(33) $$\begin{array}{c} E\left[J\left(\omega_{t+1}\left(s_{1},s_{2}\right)\right)-J\left(\omega^{*}\right)\right]\leq F^{t}E\left[J\left(\omega_{0}\right)-J\left(\omega^{*}\right)\right]\\ +\frac{2\vartheta_{1}}{LD}\left(\sum_{n=1}^{N_{1}}\sum_{i=1}^{R^{V}}D_{n}\left(1-r_{n,i}^{V}\right)+\sum_{n=N_{1}+1}^{N}\sum_{i=1}^{R^{D}}D_{n}\left(1-r_{n,i}^{D}\right)\right)\frac{1-F^{t}}{1-F}, \end{array}$$

where $F=1-\frac{\mu }{L}+\frac{4\mu \vartheta_{2}}{LD}\left(\sum_{n=1}^{N_{1}}\sum_{i=1}^{R^{V}}D_{n}\left(1-r_{n,i}^{V}\right)+\sum_{n=N_{1}+1}^{N}\sum_{i=1}^{R^{D}}D_{n}\left(1-r_{n,i}^{D}\right)\right)$. From (33), we can observe that when F<1, $F^{t}$ approximates to 0 as *t* increases. Therefore, $E\left[J\left(\omega_{t+1}\left(s_{1},s_{2}\right)\right)-J\left(\omega^{*}\right)\right]=\frac{1}{D}\left(\sum_{n=1}^{N_{1}}\sum_{i=1}^{R^{V}}D_{n}\left(1-r_{n,i}^{V}\right)+\sum_{n=N_{1}+1}^{N}\sum_{i=1}^{R^{D}}D_{n}\left(1-r_{n,i}^{D}\right)\right)\frac{1}{1-F}$ and the FL algorithm converges. When $\frac{4\mu \vartheta_{2}}{LD}\left(\sum_{n=1}^{N_{1}}\sum_{i=1}^{R^{V}}D_{n}\left(1-r_{n,i}^{V}\right)+\sum_{n=N_{1}+1}^{N}\sum_{i=1}^{R^{D}}D_{n}\left(1-r_{n,i}^{D}\right)\right)<\frac{\mu }{L}$, F<1. As $\frac{1}{D}\left(\sum_{n=1}^{N_{1}}\sum_{i=1}^{R^{V}}D_{n}\left(1-r_{n,i}^{V}\right)+\sum_{n=N_{1}+1}^{N}\sum_{i=1}^{R^{D}}D_{n}\left(1-r_{n,i}^{D}\right)\right)\leq 1$, we only need to let $\vartheta_{2}<\frac{1}{4}$. $\vartheta_{2}$ can satisfy this condition, as $\vartheta_{2}$ can be any value that satisfies $\vartheta_{2}\geq 0$. This completes the proof. □

From Lemma 3, we can also observe there is a gap $\frac{2\vartheta_{1}}{LD}(\sum_{n=1}^{N_{1}}\sum_{i=1}^{R^{V}}D_{n}\left(1-r_{n,i}^{V}\right)+\sum_{n=N_{1}+1}^{N}\sum_{i=1}^{R^{D}}$$D_{n}\left(1-r_{n,i}^{D}\right))$ between $E[J\left(\omega_{t+1}\left(s_{1},s_{2}\right)\right)]$ and $E[J\left(\omega^{*}\right)]$. As the number of participated users increases, the gap decreases. Meanwhile, as the number of users increases, the value of *F* also decreases, which improves the convergence speed of the FL algorithm.

(2) *Implementation Analysis*: Then, we analyze the implementation of the proposed algorithm. To find the optimal user selection set S, the BS must first calculate the total delay and the energy consumption $E_{n,C}^{M}+E_{n}^{P}$ of each user. In our system, the total delay includes the RF link delay $t_{n,C}^{D}+t_{n,C}^{U}+t_{n}^{P}$ and the VLC link delay $t_{dn,C}+t_{n,C}^{U}+t_{n}^{P}+t_{d,C}$. In order to calculate the total delay, the BS must know the model size required by FL algorithm and the computational time. The size of the FL model depends on the learning task and the sparsity rate. Before implementing an FL algorithm, the BS must first transmit the task information and model information to each user and set model sparsity rate. Therefore, the BS will know the FL model size and sparsity rate before training. In order to calculate the energy consumption and the computational time, the BS also needs to know the users’ device information such as transmit power and CPU. In an FL algorithm, the BS can learn the device information when users initially connect to the BS. Given the total delay $t_{n,C}^{D}+t_{n,C}^{U}+t_{n}^{P}$, $t_{dn,C}+t_{n,C}^{U}+t_{n}^{P}+t_{d,C}$, and the energy consumption $E_{n,C}^{M}+E_{n}^{P}$, the BS can compute $S_{1}$ and $S_{2}$ using **GetS**($B^{U}$,$B^{D}$,*B*). Given $S_{1}$ and $S_{2}$, the BS can compute the bandwidth of each RB using **GetB**(S). As the function in (20) is linear, the USBA-MC algorithm can determine a user selection set S to improve FL training loss.

(3) *Complexity Analysis*: With regards to the complexity of the USBA-MC algorithm, we first analyze the complexity of the model compression algorithm. In the model compression, the complexity depends on the number of model parameters. Let $W_{O}$ be the number of model parameters, the complexity of the model compression algorithm is $O(W_{O}\log W_{O})$ [26]. Then, we analyze the complexity of the traversal algorithm. Since the total number of users is N, the complexity of the traversal algorithm is O(N) [27]. In addition, the complexity of the numerical method is O(1) since we only need to allocate RBs according to the user set. Assume that the number of global iterations is *T*, and the total complexity of the USBA-MC algorithm can be expressed as $O(TW_{O}\log W_{O})$.

## 5. Simulation Results and Analysis

Consider a circular network area having a radius r=50 m with one BS at its center. There are N=50 uniformly distributed users, and 80% of the users are in indoors and 20% of them are in outdoors. The system specifications are summarized in Table 1. The following two baselines are considered: (a) the USBA algorithm in a hybrid VLC/RF system [28] and (b) the FL algorithm in RF-only system. To comprehensively evaluate the performance of the proposed USBA-MC algorithm in federated learning systems, we conduct experiments related to three learning tasks: (a) the prediction task of housing price, (b) identification task of identifying the handwritten digits from 0 to 9, and (c) identification task of classifying 10 categories of color images.

In the housing price prediction task, our goal is to compare the performance of the proposed USBA-MC algorithm under different sparsity rates, and compare the performance of USBA-MC, baselines (a) and (b). The dataset used to train the FL algorithm is Boston house price dataset (http://lib.stat.cmu.edu/datasets/boston (accessed on 27 March 2021)) that is randomly allocated to users equally. In this task, each user trains an FNN with one hidden layer composed of 10 neurons.

In the identification task of handwritten digits, we train FNNs using MNIST dataset [29]. The size of neuron weight matrices are 784 × 200, 200 × 200, and 200 × 10. Sixty-thousand handwritten digits are used to train the network and 10,000 handwritten digits are used to test it.

Finally, we train CNNs on CIFAR-10 [30] to investigate the performance of USBA-MC algorithm with different sparsity rates on non-IID data. The size of neuron weight matrices are 5 × 5 × 3 × 64, 5 × 5 × 64 × 64, 2304 × 384, 384 × 192 and 192 × 10. Fifty-thousand images are used to train the network and 10,000 images are used to test it.

### 5.1. Performance over Different Sparsity Rates

**Figure 2:**
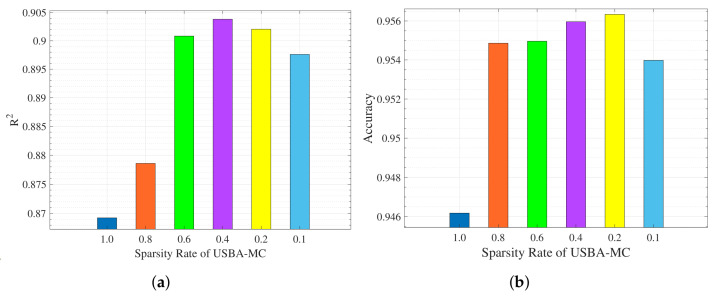
The accuracy achieved by different sparsity rates of USBA-MC in the Boston housing dataset and MNIST dataset. (**a**) Boston housing dataset. (**b**) MNIST dataset.

Figure 2 shows the performance of the proposed USBA-MC algorithm in two learning tasks under different sparsity rates. We use the coefficient of determination ($R^{2}$) to measure the quality of the model in the task of predicting housing price, and use the accuracy of classification to measure the performance in the task of identifying handwritten digits. Moreover, we calculated the average of 10 experiments to ensure the reliability of the experimental results. It shows that the $R^{2}$ values first increase and then decrease with the sparsity rate. This is because the model information will be lost with low sparsity rate. In particular, the best sparsity rate is 0.4 for predicting housing price and 0.2 for identifying handwritten digital.

**Figure 3:**
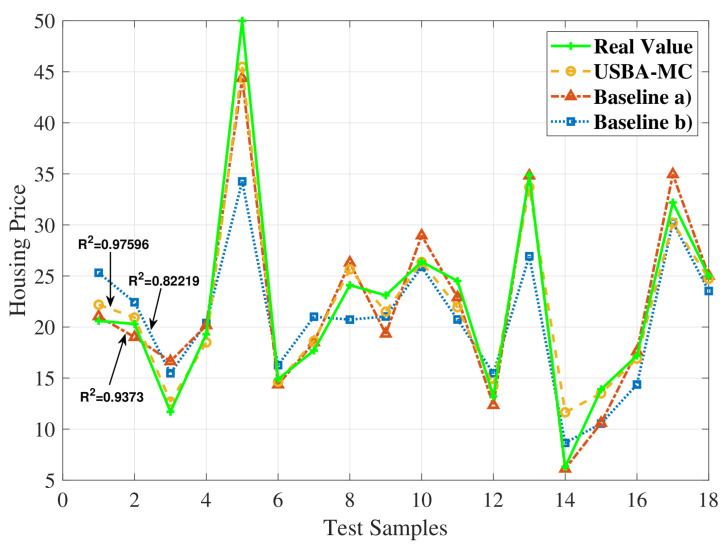
Comparison of accuracy in the Boston housing dataset trained by a BP neural network.

Figure 3 compares the predictive performance of USBA-MC, baselines (a) and (b). The green line is the true values of data samples, and the sparsity rate of USBA-MC is set to 0.4. Before training, we randomly select 18 samples to form a test set for testing. In Figure 3, we can observe the proposed USBA-MC algorithm can achieve better performance than baselines (a) and (b). In particular, the proposed FL algorithm can improve the $R^{2}$ by up to 11% and 15%, compared to baselines (a) and (b).

**Figure 4:**
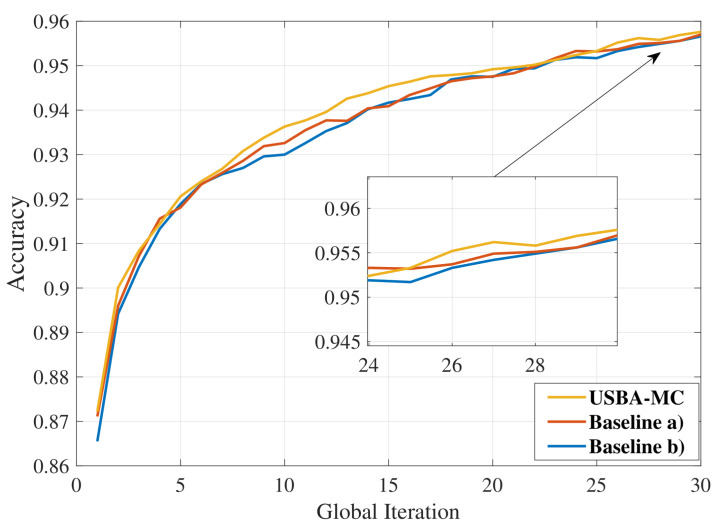
Comparison of accuracy in MNIST dataset trained by BP neural network.

Figure 4 compares the identification performance in the tasks of identifying handwritten digits. It shows USBA-MC is better than baselines (a) and (b) in most global communication rounds, and the final accuracies of these algorithms are 96.52%, 96.45%, and 96.39%, respectively. This is because USBA-MC introduces visible light communication and reduce the size of transmission model, which can increase the number of selected users, and further improving the FL performance.

### 5.2. Number of Selected Users

This subsection evaluates the performance of USBA-MC in user selection. We first compare the number of users selected under different bandwidth conditions and resource constraints.

**Figure 5:**
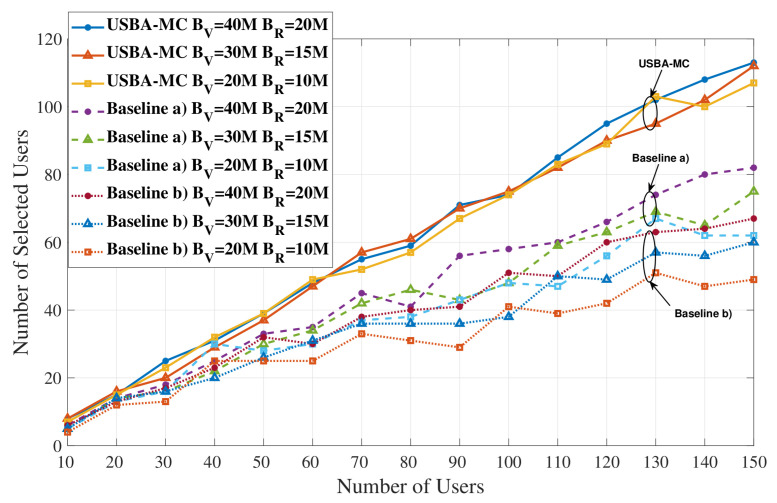
Comparison of user selection under different bandwidth settings with different numbers of users.

Figure 5 shows how the number of selected users changes as the total number of users varies in different systems. It can be observed that with the increase of the total users, the selected users in three algorithms will also increase. However, compared with baselines (a) and (b), USBA-MC algorithm enables more users to participate in the training process. This trend is more obvious with the increase of total number. For instance, when the total number is 150, the USBA-MC can improve the number of selected users, by, respectively, up to 37.8% and 68.7% compared to baseline (a) and (b). Table 2 shows the ratio of selected users, we can find that USBA-MC always has the highest ratio in all cases. Figure 5 also compares the user selection under different VLC and RF bandwidths. It can be observed that the proposed USBA-MC algorithm is better than baselines (a) and (b) under all bandwidth settings.

**Figure 6:**
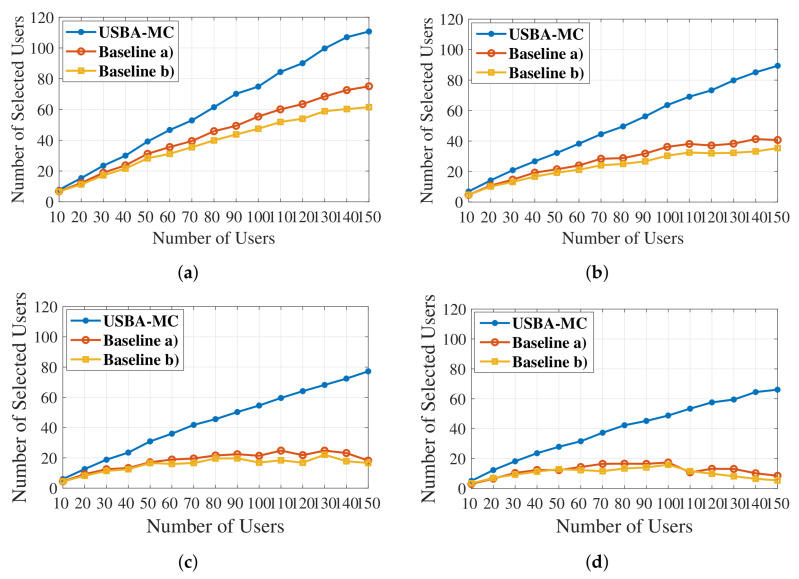
Comparison of user selection under different transmission data sizes with different numbers of users. (**a**) Data Size of 1 M. (**b**) Data Size of 3 M. (**c**) Data Size of 5 M. (**d**) Data Size of 7 M.

Figure 6 compares number of selected users under different transmission data sizes. We can observe that the selected users decrease when the data size increases. Table 3 shows the selection ratio of different algorithms with different data sizes when the total user number is 150. The result shows that USBA-MC can achieve better system performance than the other algorithms. This advantage is important when the model size becomes larger due to the complex neural network.

### 5.3. Non-IID Data

In this subsection, we explore the accuracy of USBA-MC algorithm with non-IID data [31]. To obtain a non-IID dataset, we use the same method as in [31].

**Figure 7:**
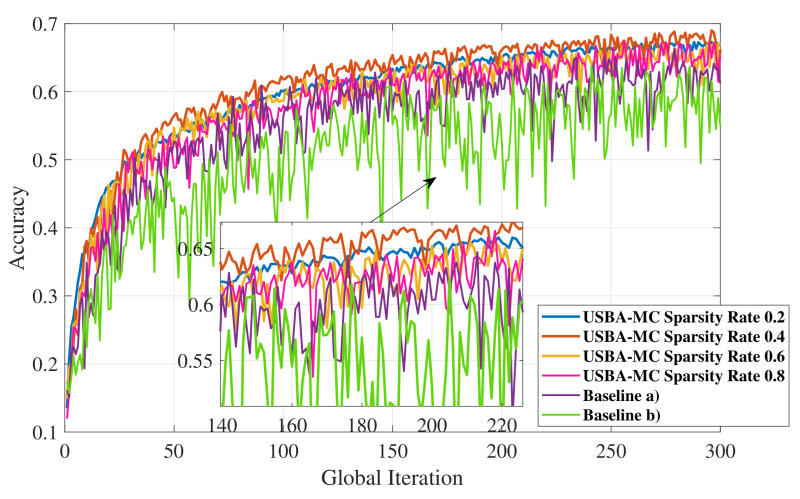
The accuracy of USBA-MC with different sparsity rates, baseline (a) and (b) on CIFAR-10 with non-IID nature.

As shown in Figure 7, the model is trained on the dataset of non-IID nature. We can clearly observe the advantages of USBA-MC compared with other algorithms. In terms of stability and accuracy, the USBA-MC algorithm achieves the best performance. In USBA-MC, a low sparsity rate will increase the stability of the system and improve the final accuracy. However, when the sparsity rate is 0.2, USBA-MC has lower accuracy and higher stability compared to 0.4. This is because decreasing sparsity rate will increase the loss of model information. Moreover, the model will not converge when the sparsity rate is too low. Therefore, there is a trade-off between the sparsity rate and the model performance.

**Figure 8:**
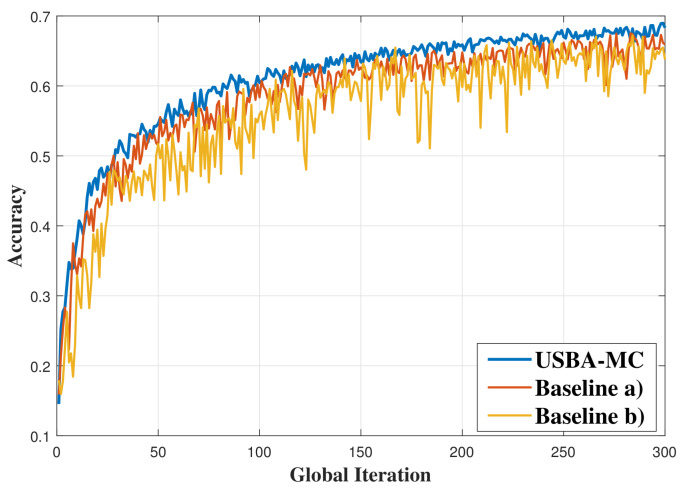
Comparison of accuracy in CIFAR-10 dataset with broadcast channel.

Figure 8 shows the use of the proposed USBA-MC algorithm for image identification. In this simulation, the BS uses broadcast techniques to transmit the global model and the local models are trained by CIFAR-10. As shown in Figure 8, the proposed USBA-MC algorithm can still achieve the best performance among the three algorithms in terms of both accuracy and stability. In particular, the USBA-MC can improve the accuracy by up to 3.27% and 6.35%, compared to baselines (a) and (b).

We analyze the gain of USBA-MC when the data set is non-IID. According to Section 3 in [31], the weight divergence will reduce the accuracy in non-IID dataset. The weight divergence is caused by the distance between the data distribution on each user and the population distribution. Such distance can be evaluated with the earth mover’s distance (EMD). According to the central limit theorem (CLT) of normal distribution [32], as the number of local models increases, the mean of EMD will be approximated by a normal distribution. Therefore, the weight divergence of the trained global model will be smaller and the model performance will be better with the increase of the number of users. Compared with the accuracy of the transmission model, the system is more sensitive to the number of selected users. Therefore, the increase of users will improve the robustness and accuracy of the global model.

**Figure 9:**
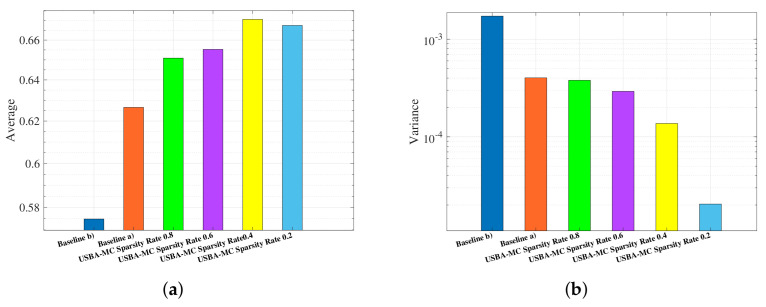
The average and variance of accuracy of USBA-MC with different sparsity rates, baselines. (**a**) Average of accuracy. (**b**) Variance of accuracy.

Figure 9 selects the model accuracy of the last 10 global communications to obtain the average and variance of the accuracy. It can be observed that the accuracy first increase and then decrease with the sparsity rate. The USBA-MC can improve the accuracy by up to 7% and 16.7%, compared to baselines (a) and (b) when the sparsity rate is 0.4. We can also observe that as the number of users increases, the model will be more stable until it cannot converge.

## 6. Conclusions

This paper has proposed the introduction of VLC into conventional RF systems for supporting FL. We have formulated a joint user selection and bandwidth allocation problem for FL in a hybrid VLC/RF system. To solve this problem, we first used a model compression method to reduce the size of FL model parameters that are transmitted over wireless links, and then we separated the optimization problem into two subproblems. The first subproblem is a user selection problem with a given bandwidth allocation, which is solved by a traversal algorithm. The second subproblem is a bandwidth allocation problem with a given user selection, which is solved by a numerical method. The convergent solution is obtained by iteratively compressing the model and solving these two subproblems. Simulation results have demonstrated that the USBA-MC algorithm outperforms USBA and FL in RF-only systems.

## Figures and Tables

**Table 1:** Simulation parameters.

Parameter	Value
Transmitted optical power per VLC AP, $P_{v}$	9 W
Modulation bandwidth for LED lamp, *B*	40 MHz
The physical area of a PD, $A_{p}$	1 cm${}^{2}$
Half-intensity radiation angle, $\theta_{1/2}$	60 deg.
Gain of optical filter, $T_{s}\left(\theta \right)$	1.0
Receiver FOV semi-angle, $\Theta_{F}$	90 deg.
Refractive index, *n*	1.5
Optical to electric conversion efficiency, γ	0.53 A/W
Noise power spectral density, $N_{0}^{VLC},N_{0}^{RF}$	$10^{-21}$ A${}^{2}$/Hz
RF total bandwidth, $B_{R}$	20 MHz
Transmit power of BS, $P_{B}$	1 W
The number of users, *N*	50
Delay requirement, $T_{round}$	2.5 s
Energy consumption requirement, $\gamma_{nE}$	2 J
Energy consumption coefficient, α	$2\times 10^{-28}$
user update size, *s*	1 Mb

**Table 2:** Selection ratio of users with different total numbers.

Total Number of User	USBA-MC	Baseline (a)	Baseline (b)
50	78%	66%	64%
100	74%	58%	51%
150	75.33%	54.67%	44.67%

**Table 3:** Selection ratio of users with different data sizes.

Data Size	USBA-MC	Baseline (a)	Baseline (b)
1 M	73.8%	50.07%	41%
3 M	59.6%	27.13%	23.6%
5 M	51.47%	12.13%	11.07%
7 M	44.07%	5.67%	3.6%

## Data Availability

Publicly available datasets were analyzed in this study. These data can be found here: http://lib.stat.cmu.edu/datasets/boston, http://yann.lecun.com/exdb/mnist, and http://www.cs.toronto.edu/kriz/cifar.html (accessed on 27 March 2021).
